# FGF19‐Activated Hepatic Stellate Cells Release ANGPTL4 that Promotes Colorectal Cancer Liver Metastasis

**DOI:** 10.1002/advs.202413525

**Published:** 2024-12-24

**Authors:** Xueying Fan, Baoting Li, Fan Zhang, Meng Liu, Hiu‐Yee Kwan, Zhongqiu Liu, Tao Su

**Affiliations:** ^1^ State Key Laboratory of Traditional Chinese Medicine Syndrome Guangdong Key Laboratory for Translational Cancer Research of Chinese Medicine International Institute for Translational Chinese Medicine Guangzhou University of Chinese Medicine Guangzhou Guangdong 510006 China; ^2^ Chinese Medicine Guangdong Laboratory Hengqin Guangdong 519031 China; ^3^ Centre for Cancer and Inflammation Research School of Chinese Medicine Hong Kong Baptist University Hong Kong 999077 China; ^4^ Institute of Research and Continuing Education Hong Kong Baptist University Shenzhen 518000 China; ^5^ Institute of Systems Medicine and Health Sciences Hong Kong Baptist University Hong Kong 999077 China

**Keywords:** ANGPTL4, cancer‐associated fibroblasts, colorectal cancer liver metastasis, FGF19, tumor microenvironment

## Abstract

Liver and lung are the most common metastatic sites in colorectal cancer (CRC), where the tumor microenvironment (TME) plays a crucial role in the progression and metastasis of CRC. Understanding the interactions between various types of cells in the TME can suggest innovative therapeutic strategies. Using single‐cell RNA sequencing (scRNA‐Seq) and clinical samples, fibroblast growth factor‐19 (FGF19, rodent FGF15) is found to mediate a significant interaction between CRC cells and cancer‐associated fibroblasts (CAFs), activating the hepatic stellate cells (HSCs)‐to‐CAFs differentiation. In various CRC metastatic mouse models, it is shown that FGF15 has a more pronounced effect on liver metastasis compared to pulmonary metastasis. More importantly, the differentially expressed genes (DEGs) are also identified from the RNA‐Seq dataset upon the activation of HSCs by FGF19 and compared the DEGs in matched primary and metastatic mRNA samples from patients with CRC liver metastasis (CRCLM), it is found that the ANGPTL4 gene is significantly associated with HSCs activation. Different mouse models also demonstrated the impact of the FGF19/ANGPTL4 axis on the severity of CRCLM. Importantly, disruption of this axis significantly inhibits CRCLM in vivo. This study is among the first to demonstrate the impact of the FGF19/ANGPTL4 axis on CRCLM, offering a novel therapeutic strategy.

## Graphical abstract

Colorectal cancer (CRC) cells exhibit the closest interaction with cancer‐associated fibroblasts (CAFs) within the tumor microenvironment (TME) of liver metastatic niches. FGF19/15 activates the hepatic stellate cells (HSCs)‐to‐CAFs differentiation, having a more significant impact on CRC liver metastasis than on pulmonary metastasis. Consequently, the FGF‐activated HSCs release angiopoietin‐like 4 (ANGPTL4) which further promotes CRC liver metastasis. Understanding the interactions between different cell types in the TME is crucial essential for developing innovative therapeutic strategies to treat CRC liver metastasis.

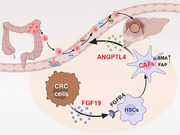

## 1 Introduction

Metastasis of colorectal cancer (CRC) presents a significant clinical challenge and remains a leading cause of cancer‐related deaths. Approximately 50% of CRC patients develop metastases, primarily in the liver and lung. Lung metastases occur in about 10%–15% of CRC patients,^[^ ^1^ ^]^ while liver metastases develop in nearly 50% of those affected.^[^ ^2^ ^]^ Among these, 15%–25% of the patients are found to have synchronous liver metastasis,^[^ ^3^ ^]^ while between 18% and 25% of the patients will develop metachronous liver metastasis within five years of their initial diagnosis.^[^ ^4^ ^]^

Treating CRC liver metastasis presents many clinical challenges. Factors such as tumor location and size, unresectable diseases, the presence of extrahepatic conditions, and the comorbidities often render surgery unsuitable for many patients. In fact, only 10%–20% of the patients are eligible for surgery intervention, resulting in a 5‐year survival rate as low as 30%. Systemic chemotherapy, biologic agents, and other adjunctive modalities have been used to target liver metastases in CRC. However, the efficacy of these treatments relies heavily on the CRC mutation status.^[^ ^5^ ^]^ Additionally, some therapeutic agents have undesirable side effects. For example, bevacizumab should be avoided in patients who are at high risk for bowel perforation or thrombotic events.^[^ ^5^ ^]^ Moreover, chemotherapy‐associated liver injury can arise from the toxicity of perioperative chemotherapy to the liver,^[^ ^5^ ^]^ and repeated cycles of systemic chemotherapy increase the risk of sinusoidal injury and hepatic insufficiency.^[^ ^6^ ^]^ These clinical challenges underscore the critical need for novel intervention strategies in managing liver metastases in CRC patients.

The process of CRC cells escaping from their primary location is complex along with many morphological changes and pathogenic phases, including the microvascular phase, extravasation and pre‐angiogenic phase, angiogenic phase, and growth phase.^[^ ^7^ ^]^ During these phases, CRC cells interact with immune cells within the liver microenvironment,^[^ ^8^ ^]^ and many of these interactions occur during the angiogenic phase and growth phase. Initially, cancer cells have to migrate to the space of Disse during the extravasation and pre‐angiogenic phases, where they recruit hepatic stellate cells (HSCs).^[^ ^9^ ^]^ Activation of HSCs is essential in the metastasis process. HSCs deposit collagen and fibronectin, forming a scaffold that facilitates the extravascular micro‐metastases of cancer cells.^[^ ^7^, ^9^ ^]^ Activated HSCs also trigger the formation of neutrophil extracellular traps in the liver that serve as metastatic niches.^[^ ^10^ ^]^ In addition, studies have shown that activated HSCs increase the expression of fibroblast activation protein alpha (FAPα) and the secretion of chemokine CXCL5. These factors promote epithelial–mesenchymal transition, which contributes to bevacizumab resistance in the CRC liver metastasis (CRCLM) treatments.^[^ ^11^ ^]^ Thus, studying the direct interaction between CRC cells and HSCs may suggest an effective novel therapeutic strategy. A recent report demonstrated that CRC‐derived exosomal miR‐181a‐5p activates HSCs and hence facilitates CRCLM.^[^ ^12^ ^]^ Previously, we reported that liver metastasis can be facilitated by the interplay between CRC cells and HSCs through the vascular endothelial growth factor (VEGF)‐interleukin 6 (IL6)‐signal transducer and activator of transcription 3 (STAT3) axis.^[^ ^13^ ^]^ However, besides the CRC cells, VEGF can also be released by various other cell types, including endothelial cells, macrophages, platelets, keratinocytes, and renal mesangial cells. Moreover, there are limitations to using anti‐VEGF therapy in cancer therapy, as cancer cells often produce additional VEGF‐A to counteract the effect of the therapy.^[^ ^14^ ^]^

Fibroblast growth factor 19 (FGF19) and its murine ortholog fibroblast growth factor 15 (FGF15) are hormonal fibroblast growth factors (FGFs) that are normally secreted into the bloodstream to exert their biological functions. These factors have been reported to be significantly upregulated in a variety of tumors.^[^ ^15^ ^]^ Typically, FGFs exhibit paracrine and/or autocrine activities by binding to specific cell membrane receptors that possess tyrosine kinase activity; the process is facilitated by high‐affinity heparin anchored to the endothelial surface.^[^ ^16^ ^]^ The FGF19 subfamily exhibits a weak affinity for heparan sulfate, resulting in a correspondingly low affinity for the FGF receptor (FGFR). The presence of the co‐receptor Klotho protein is essential for their activity in cells.^[^ ^17^ ^]^ β‐Klotho is expressed in adipose tissue, the liver, and the pancreas, with the liver being a major target organ for FGF19.^[^ ^18^ ^]^ Previous studies have demonstrated that FGF19 activates the PI3K/AKT signaling pathway in hepatocellular carcinoma (HCC). It regulates IGF2BP1, enhancing the expression of PD‐L1 by binding to FGFR4 and finally promoting the proliferation and invasion of HCC cells.^[^ ^19^ ^]^

Given the high prevalence and limited effective treatments for CRCLM, we employed single‐cell RNA sequencing (scRNA‐Seq) to identify the cell types that interact most closely with CRC cells with the tumor microenvironment (TME) in the liver metastatic niches. By revealing these interactions, we aimed to identify the critical mediators that facilitate the liver metastasis of CRC. Overall, understanding the interactions between different cell types in the TME is essential for developing novel therapeutic strategies to treat CRC liver metastasis.

## 2 Results

### 2.1 Clinical Samples and scRNA‐Seq Revealed that CRC Cells Have the Closest Interaction with Cancer‐Associated Fibroblasts (CAFs) which is Mediated by FGF19

**Figure 1:**
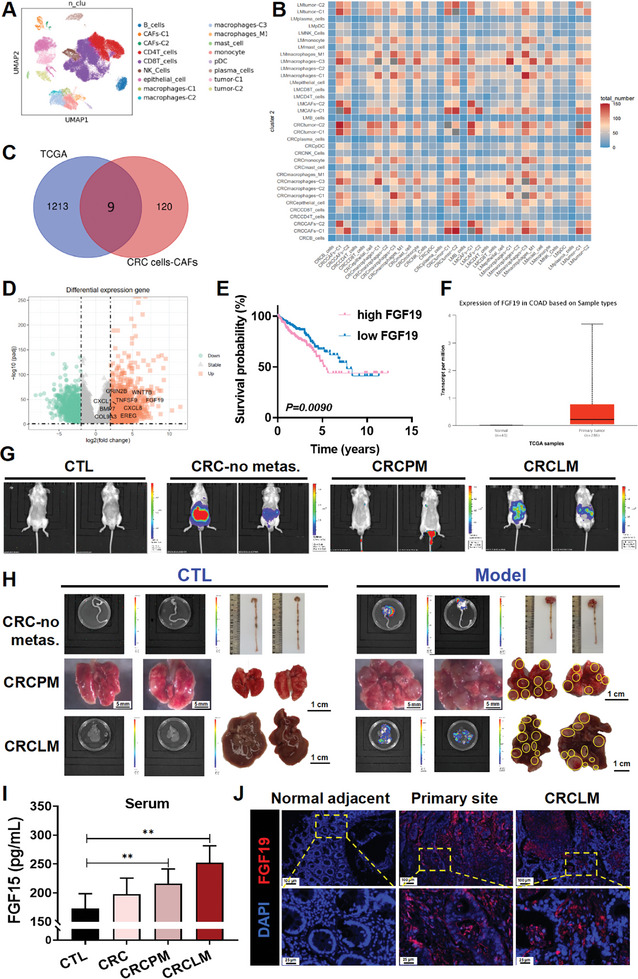
FGF19 is associated with CRC liver metastasis (CRCLM) and poor prognosis in CRC patients. A) Seventeen cell subsets were identified by analysis of the scRNA‐Seq data from 6 patients with CRCLM. B) Analysis of the cellular communication in tumor samples from patients with CRCLM. C) The Venn diagram illustrating the overlap of interacting genes between CRC cells and CAFs, as well as the highly expressed genes in CRC from the TCGA database. D) Volcano plot showing the differential expression of genes, and nine of the interacting genes exhibited a marked increase in expression. E) Correlation analysis between FGF19 expression and CRC patient survival (n = 597). F) The FGF19 expression in colon adenocarcinoma (COAD) patients from the TCGA database (Normal, n = 41; Primary tumor, n = 286). G) Representative images of the luminance signals from the CRC (nonmetastasis), CRCLM, and CRC pulmanary metastasis (CRCPM) animal models. CT26‐luciference‐expressing (CT26‐luc) cells were injected into the caecum, spleen, and tail vein of mice to establish the CRC (CRC‐no metastasis), CRCLM, and CRCPM animal models, respectively. The luminance signals were detected by using the IVIS Lumina XRMS Series III instrument at the end of the experiment. H) Representative images and the luminance signals of the colon, lung, and liver tissues of the mice in CTL (n = 7), CRC (CRC‐no metastasis) (n = 6), CRCPM (n = 7), and CRCLM (n = 7) animal models. I) The levels of FGF15 in the serum of CRC, CRCLM, or CRCPM model mice were detected using the ELISA assay (CTL, n = 7; CRC, n = 6; CRCPM, n = 7; CRCLM, n = 7). J) IF staining showing the expression of FGF19 in the clinical samples using the microarray assay (n = 42). Data are shown as Mean ± SD. For I, ^**^p< 0.01, versus CTL.

To explore CRCLM‐related cells and genes, we first analyzed a scRNA‐Seq dataset derived from patients with CRCLM by using the dimensionality reduction analysis, we identified 17 cell subsets, including B cells, T cells, tumor cells, CAFs, macrophages, and NK cells (**Figure** **1**A). After analyzing the interactions between these 17 cell subsets, we found that CRC cells exhibited the closest interaction with CAFs (Figure 1B). A total of 129 genes that interacted with CRC cells and CAFs were identified. Intersection analysis of these 129 genes with 1222 highly expressed genes in CRC from the TCGA database led to the identification of 9 significantly upregulated genes in CRC patients (Figure 1C,D). We then conducted a correlation analysis to examine the relationship between the expression levels^[^ ^20^ ^]^ of these 9 upregulated genes and the survival of CRC patients (Figure 1E,F; Figure S1A,B, Supporting Information). Interestingly, the analysis revealed that only high expression levels of FGF19 were significantly associated with poor prognosis in CRC patients (*p* < 0.05) (Figure 1E). Moreover, we utilized tissue microarrays to assess FGF19 expression at the liver metastasis site of CRCLM patients and found that FGF19 was significantly upregulated in the CRC tissues of these patients (Figure 1J). Our data agreed with other findings that FGF19 is correlated with liver metastasis^[^ ^10^ ^]^ and lower overall survival rates of CRC patients.^[^ ^21^ ^]^

### 2.2 FGF15 Plays a More Potent Role in CRCLM than in CRC Pulmonary Metastasis (CRCPM)

Given that 10%–15% of all CRC patients develop pulmonary metastases,^[^ ^22^ ^]^ we next compared the effects of FGF19 on CRC liver and lung metastasis. We established several mouse models, including a CRCPM mouse model, a CRCLM mouse model, and a nonmetastatic CRC orthotopic mouse model (Figure 1G,H). Since rodent FGF15 and human FGF19 are orthologues,^[^ ^23^ ^]^ we measured the serum FGF15 levels in these three animal models using enzyme‐linked immunosorbent assay (ELISA). Compared to the nonmetastatic mice, the serum levels of FGF15 were significantly upregulated in both the CRCPM and CRCLM mouse models. Interestingly, we found that the levels of FGF15 in the serum were higher in CRCLM mice than in CRCPM mice (Figure 1I). This finding is among the first to suggest that FGF15 plays a more significant role in CRCLM than in CRCPM.

### 2.3 FGF19 (FGF15) is Released from CRC Cells and Activates HSCs‐to‐CAFs Differentiation

**Figure 2:**
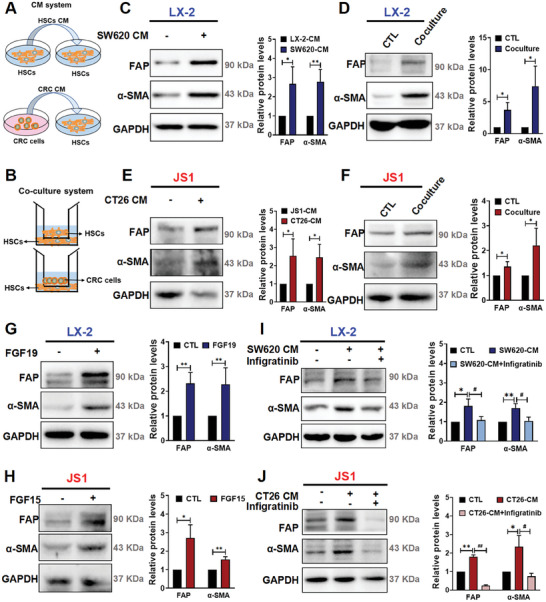
CRC cells release FGF19 (FGF15) which activates HSCs and promotes HSCs‐to‐CAFs differentiation. A) The experimental setup for the control (upper panel) and experimental (lower panel) conditions for the conditioned medium (CM) systems. B) Experimental setup for the control (upper panel) and experimental (lower panel) coculture systems. C,D) The protein levels of α‐SMA and FAP in the CM system (C) and SW620‐LX‐2 coculture system (D) were determined by the Western blotting; and the quantitative results are shown in the right panel. E,F) The protein levels of α‐SMA and FAP in the CM system (E) and CT26‐JS1 coculture system (F) were determined by the Western blotting; and the quantitative results are shown in the right panel. G) Effects of the recombinant FGF19 protein on the protein levels of FAP and α‐SMA in LX‐2 cells. H) Effects of FGF15 recombinant protein on the protein levels of FAP and α‐SMA in JS1 cells. I,J) Effects of infigratinib, a FGFR inhibitor on the protein levels of FAP and α‐SMA in SW620 CM system (I) or CT26 CM system (J). Data are shown as Mean ± SD, n = 3. For C, E: ^*^p < 0.05, ^**^p < 0.01 versus HSCs CM. For D, F‐H: ^*^p < 0.05, ^**^p < 0.01 versus the corresponding CTL. For I‐J: ^*^p < 0.05, ^**^p < 0.01 versus HSCs CM; *^#^p* < 0.05, *^##^p* < 0.01 versus CRC CM.

The TME plays a crucial role in tumor initiation, progression, and the development of metastasis. We next examined the impact of FGF19 in the TME. We first treated the human HSCs (LX‐2 cells) with conditioned medium (CM), which is collected from human SW620 CRC cells (**Figure** **2**A). We found that HSCs were activated, as evidenced by the elevated expression of CAF markers, such as alpha‐smooth muscle actin (α‐SMA) and fibroblast activation protein (FAP) (Figure 2C). To better mimic the TME, we established a coculture model of CRC cells and HSCs. Interestingly, HSCs were activated in the coculture model (Figure 2B, D). Similar results were obtained when we treated mouse HSCs (JS1 cells) with CM from CT26 mouse CRC cells or cultured CT26‐JS1 cells in the coculture model (Figure 2E,F). To determine the role of FGF19 in mediating the HSCs‐to‐CAFs differentiation, we treated HSCs with the recombinant human FGF19 protein or the recombinant mouse FGF15 protein. We found that the recombinant human FGF19 and mouse FGF15 proteins effectively activated human and mouse HSCs cells, respectively, and promoted HSCs‐to‐CAFs differentiation (Figure 2G,H). Infigratinib is a potent and selective inhibitor of FGFR1‐4.^[^ ^24^ ^]^ In the presence of infigratinib, the CM failed to trigger HSCs‐to‐CAFs differentiation (Figure 2I,J). Thus, our data strongly suggests that CRC cells release FGF19 (FGF15), which directly activates HSCs and promotes HSCs‐to‐CAFs differentiation.

### 2.4 FGF‐activated HSCs Release Angiopoietin‐Like 4 (ANGPTL4) which Increases CRC Cells Migration

**Figure 3:**
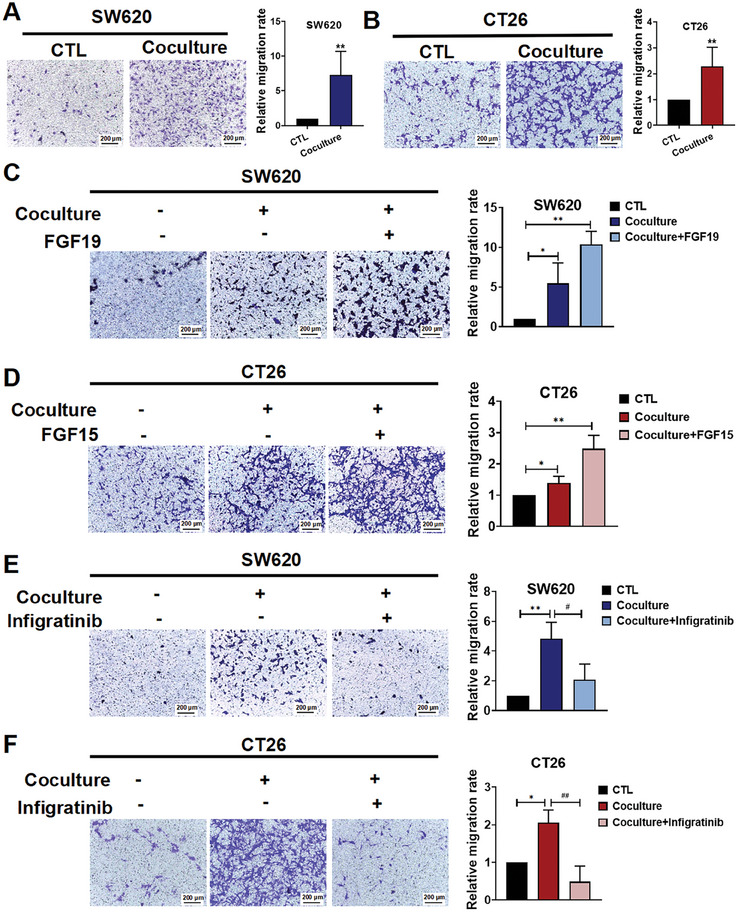
FGF‐activated HSCs increase CRC cells migration. A,B) Representative images of SW620 (A) and CT26 (B) cells migration in the CRC cells‐HSCs coculture system (left panel); and the quantitative data were analyzed using Image J software (right panel). Photographs were taken 24 h after treatment. C) Representative images of cell migration in the SW620, SW620‐LX‐2 coculture, and FGF19‐treated coculture systems (left panel); and the quantitative data were analyzed using Image J software (right panel). D) Representative images of cell migration in the CT26, CT26‐JS1 coculture, or FGF15 treated coculture system (left panel); and quantitative results were analyzed using Image J software (right panel). E) Representative images of cell migration in SW620, SW620‐LX‐2 coculture, and infigratinib‐treated coculture system (left panel); and quantitative results were analyzed using Image J software (right panel). F) Representative images of cell migration in the CT26, CT26‐JS1 coculture, and infigratinib‐treated coculture systems (left panel). All the quantitative results were analyzed using Image J software (right panel). Data are shown as Mean ± SD from three independent experiments, n = 3. For A‐D: ^*^ *p* < 0.05, ^**^ *p* < 0.01 versus the corresponding CTL. For E‐F: ^*^ *p* < 0.05, ^**^ *p* < 0.01 versus the corresponding CTL; ^#^ *p* < 0.05, ^##^ *p* < 0.01 versus CRC cells‐HSCs coculture group.

To further explore the interplay between CRC cells and activated HSCs, we cocultured CRC cells and HSCs in the coculture model and found that migration of CRC cells was significantly enhanced in this system (**Figure** **3**A,B). Furthermore, when FGF19 or FGF15 was added to the coculture, the migratory ability of the CRC cells was markedly enhanced (Figure 3C,D). To examine the interplay between CRC cells and HSCs, we included the FGFR inhibitor infigratinib in the coculture models to prevent HSCs activation by FGF19. Interestingly, infigratinib not only inhibited the activation of HSCs induced by CRC cells (Figure 2I,J) but also abolished the enhanced CRC migration observed in the coculture model (Figure 3E,F).

**Figure 4:**
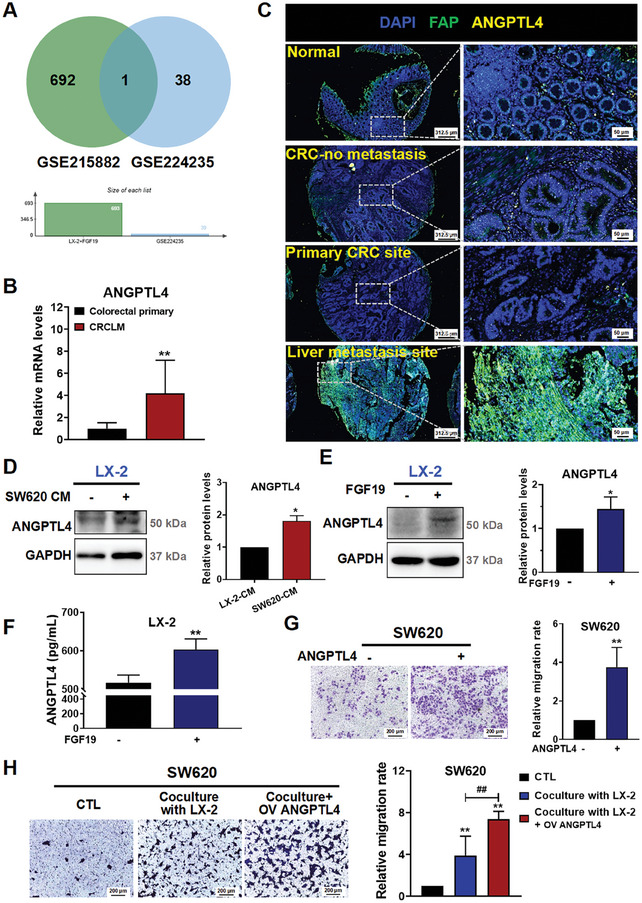
Activated HSCs secrete ANGPTL4 to promote CRC cells migration. A) Venn diagram showing the intersection between two datasets including GSE215882 and GSE224235, with ANGPTL4 was highlighted in the overlapping section. B) The mRNA levels of ANGPTL4 in tissues of the colorectal primary site and liver metastasis site were determined by spatially resolved transcriptomics (colorectal primary site, n = 9; Liver metastasis site, n = 8). C) The ANGPTL4 and FAP expression were simultaneously stained in the clinical sample tissues by using the microarray assay (n = 42). DAPI: blue color; FAP: green color; ANGPTL4: yellow color. D) The protein levels of ANGPTL4 in SW620 CM‐treated LX‐2 were determined by Western blotting (n = 3). E) Protein levels of ANGPTL4 after treated by the recombinant protein FGF19 were determined by Western blotting (n = 3). F) Levels of ANGPTL4 in the recombinant protein FGF19‐treated LX‐2 cell system (n = 4). G) Effect of the recombinant ANGPTL4 protein on the migration of SW620 cells (n = 3). H) Effect of coculture LX‐2 cells overexpressing ANGPTL4 with CRC cells on the migration ability of SW620 cells (n = 3). Data are shown as Mean ±SD from three independent experiments. For B, ^**^p < 0.01 versus the colorectal primary group. For D, ^*^p < 0.05 versus HSCs CM. For E‐G: ^*^p < 0.05, ^**^p < 0.01 versus the corresponding CTL. For H: ^**^p < 0.01 versus the corresponding CTL; ^##^ *p* < 0.01 versus SW620‐LX‐2 coculture group.

We next explored whether and how the FGF‐activated HSCs promote CRC cells migration. We analyzed an RNA‐Seq dataset GSE215882 and identified 693 differentially expressed genes (DEGs) after LX‐2 cells were activated by FGF19. We then analyzed these DEGs in the matched primary and metastatic mRNAs from 9 patients with CRCLM by conducting expression profiling using the array with accession number GSE224235. This analysis identified 39 significantly altered genes. Among them, ANGPTL4 was found to be in the interaction of these two datasets (**Figure** **4**A), suggesting that ANGPTL4 is closely related to CRCLM. Moreover, based on the GSE224235 dataset, we found that the mRNA levels of ANGPTL4 were indeed more highly expressed at the liver metastasis site in patients with CRCLM than at the primary CRC site (Figure 4B). Furthermore, the pathological role of ANGPTL4 in CRC metastasis was also confirmed by the elevated expression of ANGPTL4 in CRCLM patient tissue microarrays, as determined by the multiplex immunofluorescence (mIF) analysis (Figure 4C).

In cell models, we found that treating LX‐2 cells with SW620 CM significantly increased the protein expression of ANGPTL4 in LX‐2 cells (Figure 4D). Similarly, when LX‐2 cells were treated with the recombinant FGF19 protein, the protein expression (Figure 4E) and secretion of ANGPTL4 (Figure 4F) were also significantly increased. Our data suggest that FGF19‐activated HSCs increase ANGPTL4 expression. Given that ANGPTL4 is a secretory protein,^[^ ^25^ ^]^ it is reasonable to postulate that FGF‐activated HSCs secrete ANGPTL4, which directly affects CRC cell migration. Hence, we treated CRC cells with the recombinant ANGPTL4 protein and found that this treatment significantly increased CRC cells migration (Figure 4G). Furthermore, when SW620 CRC cells were cocultured with LX‐2 cells overexpressing ANGPTL4, the migratory capacity of SW620 cells was also significantly increased (Figure 4H). However, adding recombinant ANGPTL4 protein to SW620, or overexpressing ANGPLT4 in the coculture system did not affect the release of FGF19, indicating that ANGPTL4 does not modulate the FGF19 release (Figure S2A,B, Supporting Information). All these results strongly suggest that FGF‐activated HSCs release ANGPTL4, which increases CRC cells migration.

### 2.5 Mouse Models Demonstrate the Impact of the FGF19/ANGPTL4 Axis on the Severity of CRCLM

**Figure 5:**
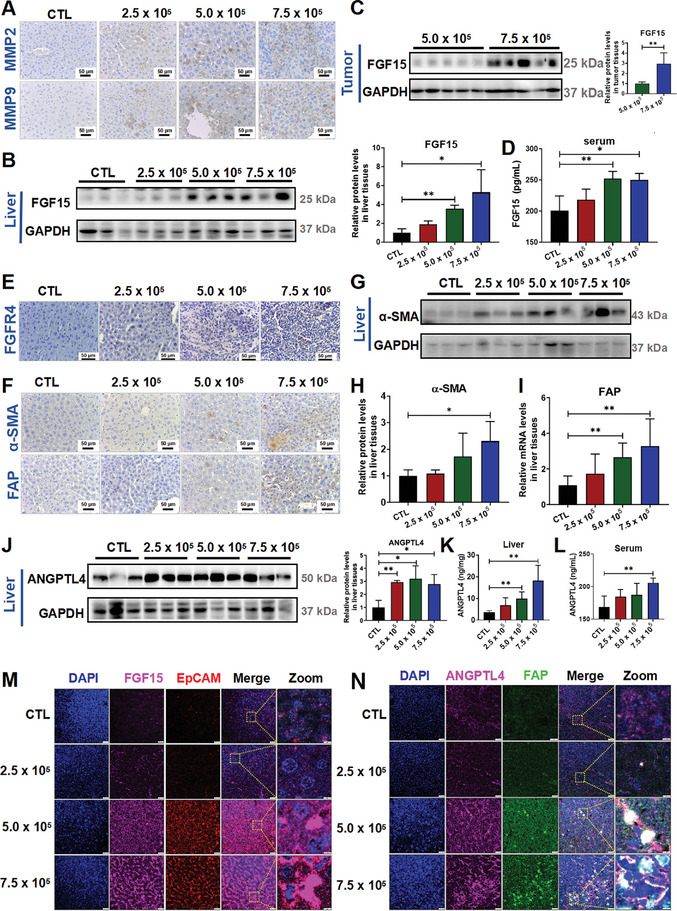
Effects of FGF15 and ANGPTL4 in mouse models of different degrees of CRCLM. A) Expression levels of MMP2 and MMP9 in the liver tissues of each group were detected by IHC staining. B,C) Protein levels of FGF15 in the liver tissues (B) and tumor sites (C) were determined by Western blotting. D) The serum level of FGF15 was determined by the ELISA assay. E) Expression of FGFR4, a FGF15 receptor in the liver tissues of each group was detected by IHC staining. F) Expression levels of α‐SMA and FAP in the liver tissues of each group were detected by IHC staining. G,H) Protein levels of α‐SMA in the liver tissues with tumors were determined by using Western blotting (G), and quantitative results were analyzed using Image J software (H). I) mRNA levels of FAP in the liver tissues with tumors were determined using RT‐qPCR analysis. J) Protein levels of ANGPTL4 in the liver tissues were determined by Western blotting (left panel), and the quantitative data were analyzed using Image J software (right panel). K,L) The liver tissue homogenates (K) and the serum (L) levels of ANGPTL4 were determined by the ELISA assay. M) Localization of FGF15 in CTCs within the liver of CRCLM mice by using the mIF analysis. N) Localization of ANGPTL4 in the liver tissue CAFs in CRCLM mouse model determined using the mIF analysis. Data are shown as Mean ± SD, n = 6. For B, D, H‐L, ^*^p < 0.05, ^**^p < 0.01 versus CTL. For C, ^**^p < 0.01 versus 5.0 × 10^5^ group.

To explore the impact of the FGF19/ANGPTL4 axis on the severity of CRCLM, we established a mouse model with varying degrees of metastasis by injecting different numbers of CT26‐luc cells (2.5 × 10^5^ cells/mice, 5.0 × 10^5^ cells/mice, 7.5 × 10^5^ cells/mice) into the spleens of BALB/c mice (Figure S3A, Supporting Information). Notably, there was no significant difference in body weight among the groups (Figure S3B, Supporting Information). Live imaging results revealed an increase in the severity of CRCLM in mice corresponding to the number of injected cells (Figure S3C–F, Supporting Information), along with gradual increases in the expression levels of matrix metalloproteinases MMP2 and MMP9 in the mouse livers (**Figure** **5**A). It is known that MMP2 and MMP9 degrade type IV collagen in the matrix, leading to the earlier formation of polymorphonuclear leukocytes (PMNs), which promote tumor metastasis.^[^ ^26^ ^]^ These observations indicated that the models had been successfully established.

Interestingly, the expression of FGF15 in both the liver (Figure 5B) and metastatic tumor tissues (Figure 5C) gradually increased with increasing extent of CRCLM in these models. The serum levels of FGF15 were also increased with the aggravation of liver metastasis of CRC (Figure 5D). More importantly, the expression level of FGF receptor 4 (FGFR4) was significantly increased along with the number of injected CRC cells (Figure 5E). In addition, more α‐SMA‐ and FAP‐positive cells were found in the mouse livers (Figure 5F). Compared to the control groups, both the protein levels of α‐SMA (Figure 5G,H) and the mRNA levels of FAP (Figure 5I) in the livers of CRCLM model mice were significantly greater, suggesting that the CAFs in the livers of the CRCLM mouse model were activated. Furthermore, the protein level of ANGPTL4 (Figure 5J) and its secretion (Figure 5K,L) were also increased with the extent of CRCLM in these models.

To further elucidate the impact of the FGF19 and ANGPTL4 on the severity of CRCLM, we analyzed their localization by using the mIF analysis. We found that FGF15 and circulating tumor cells (CTCs) colocalized in CRCLM mouse models (Figure 5M), while ANGPTL4 colocalized with CAFs (Figure 5N). These results suggested that the colocalization of these factors increased with the severity of CRCLM.

**Figure 6:**
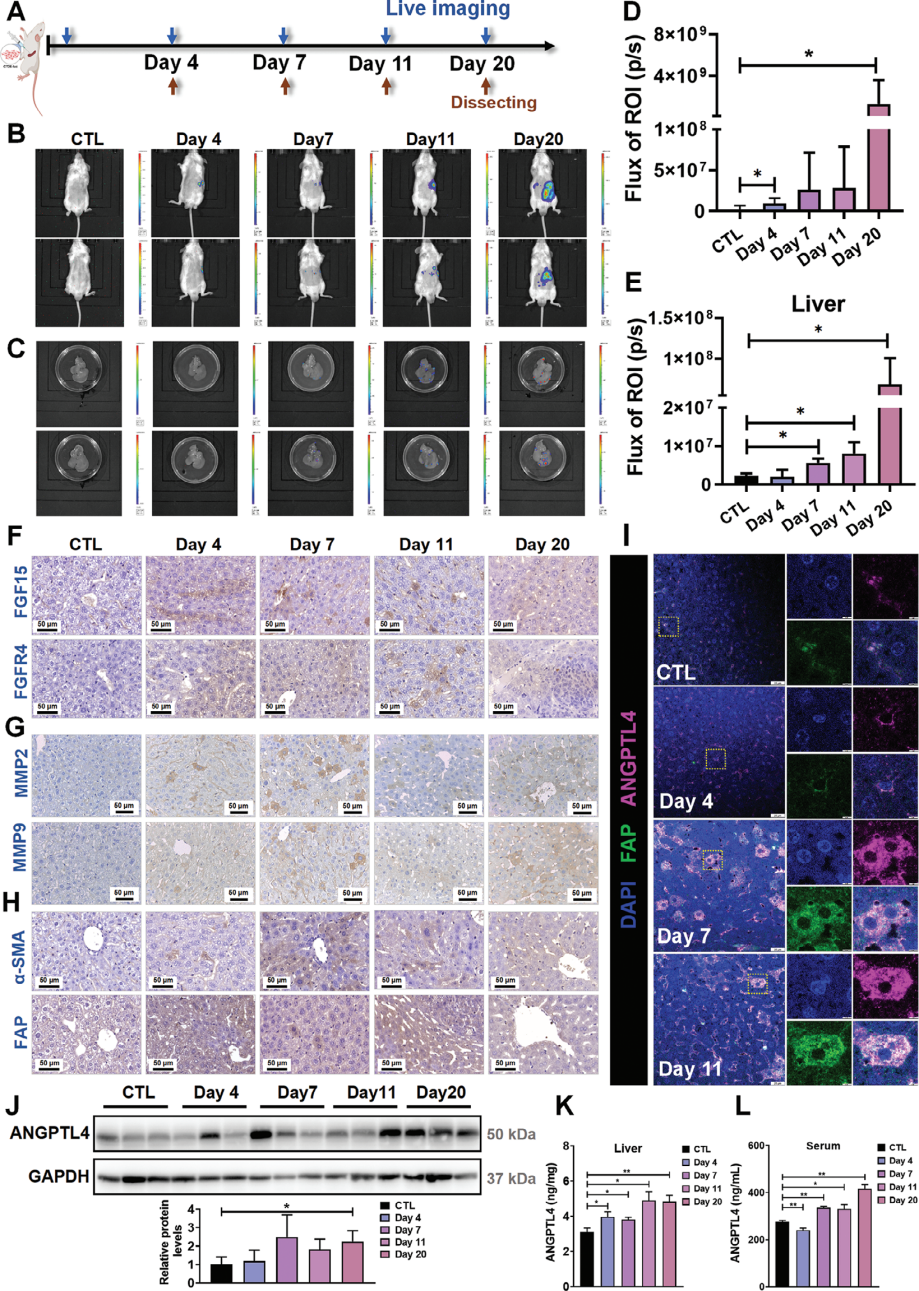
The effects of FGF15 and ANGPTL4 in CRCLM mouse models with different time points. A) Timeline for the establishment of the mouse CRCLM model. CT26‐luc cells were injected into the spleens of BALB/c mice, then the mice were sacrificed at different time points. B) Representative images of live tumor‐bearing mice with tumors. C) Representative fluorescence signal imaging of the liver tissues. D,E) Quantitative results of the mice fluorescence intensity (D) and the liver fluorescence intensity (E) were analyzed using the Living Image software 4.4. F–H) Representative IHC staining of FGF15 and FGFR4 (F) MMP2 and MMP9 (G), α‐SMA, and FAP (H) in the liver tissues of mice in each group. I) Localization of ANGPTL4 in CAFs of liver tissues in CRCLM mouse model by using the mIF analysis. J) Protein levels of ANGPTL4 in the liver tissues with tumors were determined by using Western blotting (upper panel); and quantitative results were quantified and analyzed using Image J software (lower panel). K,L) Liver tissue homogenates (K) and the serum (L) levels of ANGPTL4 were determined by the ELISA assay. Data are shown as Mean ± SD. n = 6. ^*^p < 0.05, ^**^p < 0.01 versus CTL.

In addition to establishing CRCLM model mice by injecting different numbers of CRC cells, we also established CRCLM mouse models with different molding times. We collected blood and tissue samples from the model mice on the 4th, 7th, 11th, and 20th days after cancer cells injection (**Figure** **6**A). The results showed that as the modeling time extended, the number of liver metastatic nodules increased (Figure 6B–E). Notably, there was no significant difference in body weight among the groups (Figure S4, Supporting Information). More importantly, the expression levels of FGF15 and FGFR4 in the livers of mice were increased with the extent of metastasis (Figure 6F). Moreover, CAFs were activated as evidenced by the increased expression levels of MMP2, MMP9 (Figure 6G), α‐SMA, and FAP (Figure 6H) in the liver tissues of the mice. More importantly, we also found that ANGPTL4 was predominantly expressed in the CAFs of the liver tissues (Figure 6I), and its expression level (Figure 6J) and secretion (Figure 6K,L) were also increased with the extent of metastasis. Taken together, our data clearly demonstrated the impact of FGF19 and ANGPTL4 on the severity of CRCLM.

### 2.6 Abolishing the FGF19/ANGPTL4 Axis Inhibits CRC Liver Metastasis in vivo

**Figure 7:**
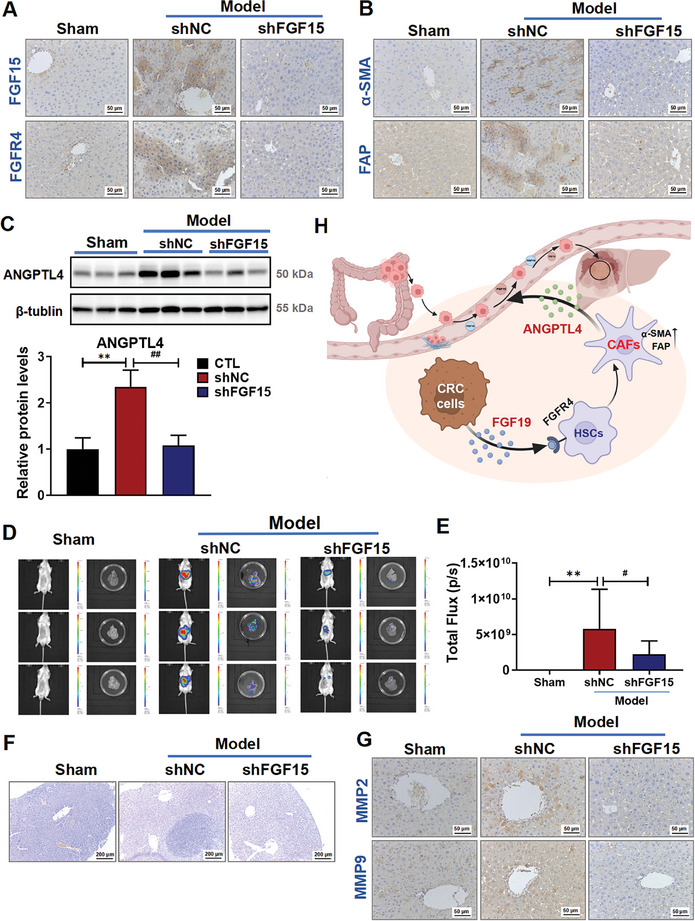
The FGF15/ANGPTL4 axis is involved in the progression of CRCLM. A,B) Representative images of IHC staining for FGF15 and FGFR4 (A), and for α‐SMA and FAP (B) in the liver tissues of mice in each group. C) The protein levels of ANGPTL4 in tumor‐bearing liver tissues were determined by using Western blotting (upper panel); and quantitative results were analyzed using Image J software (lower panel). D) Representative live‐animal imaging images of mice with tumors. CT26‐luc‐shNC cells and CT26‐luc‐shFGF15 cells were inoculated into the hepatic portal vein of BALB/c mice, respectively. Mice were randomly divided into 3 groups, including the sham, shNC, and shFGF15 groups. E) Quantitative results of the fluorescence intensity were analyzed using Living Image software 4.4. F) Representative H&E‐stained images of liver tissues in each group of mice. G) Representative images of IHC for MMP2 and MMP9 in mouse liver tissues from each group. H) Diagram showing that the FGF19/ANGPTL4 axis mediates the interaction between CRC cells and HSCs, and promotes CRC liver metastasis. Data are shown as Mean ± SD, n = 6. ^**^p < 0.01 versus Sham; *^#^p* < 0.05, *^##^p* < 0.01 versus shNC.

To further explore the impact of the FGF19/ANGPTL4 axis on CRCLM, we knocked down FGF15 in CT26‐luc cells (shFGF15) using lentiviral infection before injecting these cells into the portal vein of the mice to establish a CRCLM mouse model. Our data showed that the expression levels of FGF15 and FGFR4 were significantly lower in the shFGF15 group compared to the shNC group (**Figure** **7**A). Additionally, the expressions of two HSCs activation biomarkers, α‐SMA and FAP (Figure 7B), were also significantly reduced, suggesting that FGF15 knockdown in CRC cells reduces HSCs activation. Interestingly, we also found that the levels of ANGPTL4 in the liver were significantly reduced (Figure 7C). Furthermore, both CRC liver metastasis (Figure 7D‐E) and the size of the metastatic nodules in the livers of mice (Figure 7F), along with the expression levels of MMP2 and MMP9 in the liver (Figure 7G) were significantly reduced. There was no significant difference in the body weight among the groups (Figure S5, Supporting Information). Taken together, these findings suggest that abolishing the FGF/ANGPTL4 axis inhibits CRCLM in vivo.

## 3 Discussion

Our study demonstrated that CRC cells exhibit the closest interaction with CAFs at the liver metastatic sites, where the cancer cells secrete FGF19 to promote HSCs‐to‐CAFs differentiation. Activated HSCs, in turn, release ANGPTL4 to increase the metastatic potential of the CRC cells. This interplay between CRC cells and HSCs, along with the impact of the FGF19/ANGPTL4 axis on the severity of CRC liver metastasis, suggests a novel therapeutic strategy for treating CRC liver metastasis (Figure 7H).

The liver microenvironment is intricate. Employing scRNA‐Seq to reveal the interactions between different cell types in this microenvironment is a promising approach to further our understanding. A study mapped the TME of liver metastases from CRC using data from 111292 single cells, revealing transcriptional changes and phenotypic alterations within the TME in response to chemotherapy, particularly highlighting shifts in the abundances of B cells and tumor‐associated macrophages (TAMs).^[^ ^27^ ^]^ These findings suggest that the abundance and populations of immune cells in the microenvironment are continuously changing, which may pose challenges to the treatment. In other words, targeting the immune cells for treatments may be influenced by the temporal changes in the TME landscape. Moreover, targeting the immune environment is challenging because many cytokines and chemokines exhibit redundancy in their actions.^[^ ^28^ ^]^ This redundancy refers to the phenomenon where different cytokines and chemokines can exert similar actions. Hence, inhibiting the release of a single cytokine or chemokine for cancer treatment may yield limited efficacy.

Despite the presence of various immune cell types in the TME, our data revealed that CRC cells have the closest interaction with CAFs. Although CAFs can originate from HSCs, hepatic sinusoidal endothelial cells, and mesenchymal stromal cells,^[^ ^29^ ^]^ our data clearly demonstrated that CRC cells activate HSCs and promote HSCs‐to‐CAFs differentiation by releasing FGF19. These data suggest that CAFs identified in our study originated from HSCs.

FGF19 belongs to a family of heparin‐binding growth factors that consist of 22 members. FGF19 is one of the extracellular FGF family members that exerts its biological function by binding to high‐affinity FGFR4. FGF19‐FGFR4 signaling has been implicated in many biological processes, including metabolism.^[^ ^30^ ^]^ In human plasma, FGF19 levels are elevated postprandially through the activation of the bile acids‐farnesoid X receptor (FXR) axis, which reduces the expression of the rate‐limiting enzyme CYP7A1 in the liver, and hence controls bile acid synthesis.^[^ ^31^ ^]^

In this study, we showed that FGF19 activates HSCs‐to‐CAFs differentiation, which aligns with the findings from other studies.^[^ ^10^ ^]^ However, in patients with liver fibrosis, lower levels of FGF19 are associated with more severe hepatic fibrosis,^[^ ^32^ ^]^ suggesting an antifibrotic effect of FGF19. In fact, the benefits of direct FGF19 treatment for liver fibrosis have been demonstrated in animal models.^[^ ^33^ ^]^ Furthermore, treatment with FGF19 analogs also significantly improved liver fibrosis in patients with nonalcoholic steatohepatitis during phase II clinical trials.^[^ ^34^ ^]^ However, our coculture model data clearly showed that FGF19 released from CRC cells activates HSCs, and this activation can be reversed by infigratinib, suggesting that the activation of HSCs by FGF19 is a specific effect. It is possible to suggest that FGF19 may have dual roles depending on the presence or absence of cancer cells. Besides, the action of FGF19 also greatly depends on the downstream signaling in the HSCs that may be affected by other factors in the coculture model or in the TME in the liver. Similarly, FGF19 has been reported to have dual roles in controlling lipid levels. Although FGF19 is used to treat metabolic diseases such as nonalcoholic steatohepatitis and diabetes,^[^ ^35^ ^]^ one study indicated that FGF19 has both lipid‐raising and lipid‐lowering effects that may be mediated by different FGF receptors and target tissues.^[^ ^36^ ^]^ Our findings reveal that FGF19 activates HSCs in the CRCLM models, which may warrant further investigation into the use of FGF19 analog to treat metabolic diseases in cancer patients.

We are among the first studies to report that ANGPTL4 is released by FGF19‐activated HSCs. Physiologically, the expression of ANGPTL4 is regulated by glucocorticoids, and its well‐known functions are related to the regulation of metabolic pathways.^[^ ^25^, ^37^ ^]^ For example, ANGPTL4 inhibits lipoprotein lipase, reducing extracellular lipolysis and fatty acid uptake by adipocytes.^[^ ^37a^ ^]^ In addition, ANGPTL4 enhances the effects of catecholamines by increasing cAMP‐dependent triglyceride hydrolysis.^[^ ^37a^ ^]^ The role of ANGPTL4 in CRC has also been previously reported, it promotes glucose uptake and increases glycolysis in cancer cells.^[^ ^38^ ^]^ Indeed, the expression levels of ANGPTL4 in CRC patients are directly associated with metastasis^[^ ^39^ ^]^ and are dependent on cancer stages and hypoxic conditions.^[^ ^40^ ^]^

The development of CRC liver metastasis is complex.^[^ ^41^ ^]^ The existence of pre‐metastatic niches may have significant implications for the clinical management of metastatic disease.^[^ ^7b^ ^]^Although the resident and immune cells of the liver play diverse and opposing roles in the development of metastases, HSCs are essential for orchestrating a pro‐metastatic microenvironment.^[^ ^7b^ ^]^ Targeting the specific interactions between CRC cells and HSCs could be a promising strategy, especially considering the heterogeneity of the liver microenvironment and the spatiotemporal reprogramming of the immune cell populations during disease development.^[^ ^42^ ^]^ Additionally, it has been discovered that inhibiting the release of ANGPTL4 from HSCs or targeting FGF19 are the potential strategies. Competitive inhibition of the FGF19 receptor with the modified nontumorigenic FGF19 variant M70 is also feasible. M70 has five amino acids deleted and three amino acids substituted at the N‐terminal region. Studies have shown that M70 retains the biological function of FGF19 in reducing serum bile acid levels, as validated in phase I clinical studies.^[^ ^43^ ^]^ However, it does not promote the growth of cancers such as liver cancer.^[^ ^44^ ^]^ Whether M70 can be used to competitively inhibit the FGF19 receptor in the CRC cells‐HSCs interplay for treating CRC liver metastasis remains to be explored.

Until now, the regulatory mechanism of ANGPTL4 on CRC cells migration remains less studied. In this study, we revealed for the first time that ANGPTL4 is secreted from HSCs and enhances CRC cells migration. The liver is the central organ for fatty acid metabolism, fatty acids accumulate in the liver through hepatocellular uptake from the plasma and by *de novo* biosynthesis.^[^ ^45^ ^]^ Studies have reported that ANGPTL4 has profound effects on lipid metabolism, affecting both uptake and storage. More importantly, the knockdown of ANGPTL4 inhibits lipids production, proliferation, and invasion in lung adenocarcinoma cells.^[^ ^46^ ^]^ Cancer cells within the tumor microenvironment undergo rapid proliferation, survival, migration, and invasion through altered lipid metabolism.^[^ ^47^ ^]^ In this study, we found that ANGPTL4 promotes CRC cell migration, suggesting that the underlying mechanism may be associated with the modulation of lipid metabolism. Currently, anticancer drugs targeting metabolic pathways can be used in combination with various treatments to enhance efficacy. For example, metformin, ritonavir, or LXRα agonists affect both glucose metabolism and lipid metabolism, and these drugs are already in clinical use for other indications.^[^ ^48^ ^]^ In the future, developing drugs that target the FGF19/ANGPTL4 axis could represent an effective therapeutic strategy for treating CRCLM by regulating lipid metabolism.

Our study has shown that CRC cells exhibit the closest interaction with CAFs within the TME of liver metastatic niches. Additionally, our data clearly demonstrate the direct interaction between CRC cells and HSCs, as well as the influence of FGF19 and ANGPTL4 on the severity of CRCLM. These findings suggest a novel mechanism underlying the progression of CRCLM, which could inform the development of therapeutic strategy.

## 4 Experimental Section

### Chemicals and Reagents

Antibodies against GAPDH (Cat# sc‐32233), α‐SMA (Cat# sc‐53142), FGF19 (Cat# sc‐390621), FGF15 (Cat# sc‐514647), MMP9 (Cat# sc‐13520), FGFR4 (Cat# sc‐136988), and ANGPTL4 (Cat# sc‐373761) were purchased from Santa Cruz Biotechnology (Santa Cruz, CA, USA); antibodies against MMP2 (Cat# 87809) and goat anti‐rabbit HRP (Cat# 7074) were purchased from Cell Signaling Technology (CST, MA, USA); antibody against EpCAM (Cat# bs‐1513R) was purchased from Beijing Biosynthesis Biotechnology Co., Ltd. (Bioss, Beijing, China); antibody against FAP (Cat# A11572) was purchased from ABclonal Technology Co., Ltd. (ABclonal, Wuhan, China); goat anti‐mouse IgG HRP (Mu Biotech, Cat# 125035) was purchased from Mu Biotechnology, Inc. (Mu Biotech, Guangzhou, China). Multimer anti‐rabbit/mouse IgG HRP for immunohistochemistry (IHC) staining was purchased from Wuhan BOSTER Bioengineering Co., Ltd (BPSTER, Wuhan, China). Human FGF19 recombinant protein was purchased from PeproTech Inc. (PeproTech, NJ, USA). Infigratinib, human angiopoietin‐related 4 protein (ANGPTL4, HEK293, His), and mouse FGF15 Protein (His‐SUMO) were purchased from MedChemExpress (MCE, NJ, USA). ELISA commercial kits including FGF15 and ANGPTL4 were purchased from Quanzhou Ruixin Biotechnology Co., Ltd. (Fujian, China). The human FGF19 ELISA commercial kit was purchased from Jiangsu Meimian Industrial Co., Ltd. (Jiangsu, China). XenoLight D‐luciferin potassium salt was purchased from PerkinElmer (PKI, MA, USA). ECL detection kit was purchased from GBCBIO Technologies Inc. (GBCBIO, Guangzhou, China). TSAPLus fluorescence triple staining kit was purchased from Wuhan Servicebio Technology Co., Ltd. (Servicebio, Wuhan, China). Hematoxylin and eosin (H&E) stain solution was purchased from Beijing Labgic Technology Co., Ltd. (Biosharp, Beijing, China).

### Cell Culture

CRC cell line (SW620), and HSC (LX‐2) were purchased from Wuhan Pricella Biotechnology Co., Ltd. (Wuhan, China). CRC cell line (CT26), and HSC (JS1) were purchased from Shanghai EK‐Bioscience Biotechnology Co., Ltd. (Shanghai, China). CT26‐luc was purchased from Smart Biotechnology Co., Ltd. (Guangzhou, China).

SW620, LX‐2, and JS1 cells were cultured in high‐glucose Dulbecco's modified Eagle's medium (DMEM) containing 10% fetal bovine serum (FBS) (Gibco, USA) and 1% penicillin/streptomycin (P/S) (Biosharp, China). CT26 and CT26‐luc were cultured in RPMI 1640 containing 10% FBS (Gibco, USA) and 1% P/S (Biosharp, China).

### Patient Tissue Specimens

The human tissue microarray (TMA) was purchased from Shanghai Outdo Biotech Co., Ltd. (HColA060CD01). Patients who were diagnosed with relapse as well as those who received preoperative radiation, chemotherapy, or biotherapy were excluded. Demographic and clinical data were obtained from the patients’ medical records. Detailed patient information is presented in Table S1 (Supporting Information). All tissue samples were collected with the informed consent of the patient, and the center's Institutional Review Board approved this study.

### Lentiviral Vector Construction and Infection

The full‐length sequence or shRNA of FGF15 was constructed into the lentiviral vector (GenePharma, Suzhou, China). Infection experiments were executed according to the manufacturer's instructions. Briefly, 2 × 10^5^ CT26‐luc cells were seeded into 6‐well plates the day before transfection. The lentivirus was premixed with a complete RPMI‐1640 medium (2 mL) and subsequently added to CRC cells. After 24 h, the medium was replaced with a fresh culture medium. Stable cell lines were selected via incubation with puromycin (10 µg mL^−1^) for 7 days.

### Cell Transfection

pcDNA3.1‐NC and pcDNA3.1‐ANGPTL4 were purchased from Suzhou GenePharma Co., Ltd. (GenePharma, Suzhou, China). LX‐2 cells were seeded to 60–70% confluency and allowed to attach overnight, after that cell transfection was performed with Lipofectamine 3000 (Invitrogen, CA, USA) following the manufacturer's instructions. Subsequent experiments were performed after transfection for 48 h.

### Identification of Different Cell Types

After the dataset GSE178318 was processed for quality control, clustering, and annotation were performed using the function FindClusters with a resolution of 1.0. The annotation of each cell cluster was confirmed by the expression of typical marker genes.^[^ ^49^ ^]^

### Cell‐cell Communication Analysis

Cell Phone DB v2.0.6 (www.cellphonedb.org) was used to investigate the communication between cell types in CRCLM by entering files containing gene expression values and annotated cell type information.^[^ ^50^ ^]^

### Conditioned Medium (CM) System

The generation of CM was performed as previously described.^[^ ^13^ ^]^ Briefly, CRC cells (SW620 or CT26) were seeded in 6‐well plates and cultured in DMEM or RPMI 1640 containing 10% FBS for 24 h. Then, cells were washed twice with 1 × PBS and then cultured in FBS‐free DMEM for 24 h. Afterward, the medium was collected and centrifuged for 5 min at 1200 rpm, and then the supernatant (CM) was used to incubate with HSCs.

### Coculture System

To explore the interaction between HSCs and CRC cells, as previously described,^[^ ^13^ ^]^ HSCs were cocultured with CRC cells in two‐chamber dishes, which allowed for the exchange of soluble diffusible factors while preventing direct contact between the cell types. LX‐2 and JS1 (2 × 10^5^ cells/well) were seeded in a polycarbonate transwell membrane with 0.40 µm pores coated with collagen (LABSELECT, Hefei, China). SW620 and CT26 (4 × 10^5^ cells/well) were seeded in the lower chambers and incubated for 24 h in an FBS‐free DMEM medium.

### Migration Assay

The migration assay was performed using hanging cell culture inserts (polyethylene terephthalate (PET) membranes with 8 µm pores) (LABSELECT, Hefei, China). A total of 2.5 × 10^4^ cells (SW620 or CT26) were treated with or without ANGPTL4 (100 ng mL^−1^), FGF19/15 (50 ng mL^−1^), and then seeded in the upper chamber with 200 µL of FBS‐free DMEM. The lower chamber, seeded or not with HSCs (LX‐2 or JS1), received DMEM (800 µL) with 10% FBS and was treated with or without infigratinib (60 nM). After 24 h of incubation, the cells in the upper chamber were removed. The invaded cells at the bottom of the PET membrane were fixed with 4% paraformaldehyde and stained with crystal violet (GBCBIO, Guangzhou, China). Invaded or migrated cells were counted and imaged by a light microscope (Leica, Germany).

### Western Blotting

Total protein from cells or tissue samples was extracted using RIPA lysis buffer containing 1% PMSF and 1% phosphatase inhibitor (Beyotime, Jiangsu, China). After centrifugation at 12 000 rpm at 4 °C, the supernatant was collected for protein concentration detection using the Bradford method. After denaturation, the protein extract was resolved by 10% or 12% SDS‐PAGE and transferred to the PVDF membrane (0.45 µm, Millipore, Bedford, MA, USA). The PVDF membrane was incubated with diluted primary antibody at 4 °C overnight and then incubated with the secondary antibody at room temperature for 2 h. The immunoreactive bands were visualized using an ECL detection kit (GBCBIO, Guangzhou, China), and the Western blot bands were quantitatively analyzed by Image J.

### Quantitative Real‐time Polymerase Chain Reaction (RT‐qPCR)

Total RNA was extracted from cells or tissue samples using Trizol reagent (Vazyme, Nanjing, China). An Evo M‐MLV reverse transcription premixed kit (AG, Hunan, China) was used for cDNA synthesis, and a SYBR green premix pro tag HS qPCR Kit (AG, Hunan, China) was used for RT‐qPCR. The 2^−ΔΔCt^ method was used for the comparison between groups with GAPDH as an internal control. The qPCR primers used are listed in Table S2 (Supporting Information).

### Animal Experiments

Male BALB/c mice 5‐week‐old were purchased from the Guangdong Medical Laboratory Animal Center [SCXK(GZ)2022‐0022, Guangzhou, China] or the Laboratory Animal Center of Southern Medical University [SCXK(GZ)2021‐0041, Guangzhou, China]. All animal experiments were approved by the Institutional Animal Care and Use Committee of the International Institute for Translational Chinese Medicine, Guangzhou University of Chinese Medicine, and the mice were kept in the animal laboratory at International Institute for Translational Chinese Medicine [SYXK(GZ)2024‐0144].

**Establishment of CRC models with different sites of metastasis**: For the CRC cecum graft in situ tumor and CRCLM models, after anesthesia, the cecum or spleen of each mouse was exteriorized through a laparotomy for the CRC cecum graft in situ tumor and CRCLM models. CT26‐luc cells suspended in 50 µL PBS were injected into the plasma membrane of the cecum or the distal tip of the spleens with an insulin syringe. A whitening bulge of the cecum or spleen should be observed upon injection area. After the whitening bulge disappeared, the cecum or spleen was repositioned, and the abdominal wound was then sutured. For the CRCPM model, CT26‐luc cells suspended in 50 µL of PBS were injected into the tail vein of mice. A live imaging system IVIS Lumina XRMS Series III (PerkinElmer, MA, USA) was used to detect the in vivo liver metastases. Image analyses were performed using the Living Image software 4.4.
**Establishment of CRC models with different degrees of CRCLM**: BALB/c mice were injected intrasplenically with no cells or 2.5 × 10^5^ cells/50 µL, 5.0 × 10^5^ cells/50 µL or 7.5 × 10^5^ cells/50 µL of CT26‐luc cells under isoflurane anesthesia. To observe the formation of cancer metastases formation and tumor growth, mice received an intraperitoneal injection of 150 mg kg^−1^ XenoLight D‐Luciferin potassium salt once every five days and were imaged using the IVIS Lumina XRMS Series III (PerkinElmer, MA, USA). Image analyses were performed using the Living Image software 4.4.
**Establishment of CRC models with different stages of CRCLM**: BALB/c mice were injected intrasplenically with 5.0 × 10^5^ cells/50 µL CT26‐luc cells under isoflurane anesthesia. Living Image software was used to monitor the extent of tumor metastasis in mice. Mice were euthanized at the following time points: no metastasis (Day 4), mild metastasis (Day 7), moderate metastasis (Day 11), or severe metastasis (Day 20).
**Establishment of the shFGF15 of CRCLM Model**: To investigate the effect of FGF15 on CRCLM in vivo, three groups of randomly selected BALB/c mice were injected in hepatic portal vein with 5.0 × 10^5^ sh‐NC or sh‐FGF15‐infected CT26‐luc cells suspended in PBS, while sham mice received PBS via hepatic portal vein with PBS. Living Image software was used to monitor the extent of tumor metastasis in the mice.

### Hematoxylin and Eosin (H&E) and Immunohistochemical (IHC) Staining

The resected liver samples were fixed in 4% paraformaldehyde and paraffin‐embedded by routine processing. Sections were cut to a thickness of 4 µm, heated at 60 °C for 60 min, and then deparaffinized and hydrated with a series of xylene and alcohol solutions. H&E staining was performed, followed by dehydration. Images were subsequently obtained with a histopathology slide scanner (Leica, Germany). After deparaffinization and rehydration, the slides were microwaved for antigen retrieval. The sections were then rinsed with PBS and immersed in 3% H_2_O_2_ to block endogenous peroxidase activity. Subsequently, the sections were incubated with 10% BSA to block the nonspecific binding. IHC staining was performed using anti‐α‐SMA (1:200), anti‐FAP (1:200), anti‐FGF15 (1:200), anti‐FGFR4 (1:200), anti‐MMP2 (1:200) or anti‐MMP9 (1:200) antibodies at 4 °C for overnight. After incubation with the horseradish peroxidase (HRP)‐conjugated secondary antibody polymer for 2 h, the images were visualized with a histopathology slide scanner (Leica, Germany).

### Immunofluorescence (IF) Staining

The TSAPLus fluorescence triple‐staining kit obtained from Servicebio (Wuhan, China) was used for multiple fluorescent immunolabeling of multiple antigens in the liver sections according to the manufacturer's protocols. The TMA TSAPLus fluorescence triple staining was performed by Hunan Aifang Biotechnology Co., Ltd. (Hunan, China). The degree of staining was scored as described below. Briefly, the TMA was deparaffinized, heat treated in antigen retrieval buffer, then blocked and incubated with primary antibody. This was followed by the application of HRP‐conjugated secondary antibody polymer, using TSA chemistry to deposit dyes on the tissue surrounding each HRP molecule. The nuclei were counterstained with DAPI dye. Finally, the TMA was mounted with a fluorescence mounting medium and was examined using a fluorescence microscope. Images were acquired with a KFBIO KF‐FL‐020 digital pathology scanner.

### ELISA Assay

The levels of FGF15 and ANGPTL4 in the serum and liver tissues and the levels of FGF19 and ANGPTL4 in the cell culture supernatant were determined by using the commercial ELISA kits according to the manufacturer's instructions.

### Statistical Analysis

All quantitative data were presented as Mean±standard deviation (SD). The samples or mice per group in each study are marked in the related figure legend (n). All data were analyzed statistically using IBM SPSS Statistics 26 software. LSD was used for multiple comparisons when the variances were in agreement, while Tamhane's test was used for the test when the variances were not in agreement. A *p*‐value of < 0.05 was considered statistically significant. All experiments were plotted using GraphPad Prism 8.0 software.

## Conflict of Interest

The authors declare no conflict of interest.

## Author Contributions

F.X.Y. and L.B.T. performed the experiments. Z.F. and L.M. helped to complete the experiments. F.X.Y. and S.T. analyzed data. S.T. and K.H.Y. wrote the original manuscript. L.Z.Q. reviewed and edited the manuscript. S.T., K.H.Y., and L.Z.Q. conceived and supervised the study, and approved the final manuscript. All authors have read and approved the article.

## Supporting information



Supporting Information

## Data Availability

The data that support the findings of this study are available from the corresponding author upon reasonable request.
