# Supplementary material for: FGF19‐Activated Hepatic Stellate Cells Release ANGPTL4 that Promotes Colorectal Cancer Liver Metastasis
Source: Adv Sci (Weinh). 2024 Dec 24;12(7):2413525. doi: 10.1002/advs.202413525 (PMC11831508; doi:10.1002/advs.202413525)
Supplement: Supplementary file 1 — Supporting Information [file ADVS-12-2413525-s001.docx]

Supporting Information

**FGF19-Activated Hepatic Stellate Cells Release ANGPTL4 that Promotes Colorectal Cancer Liver Metastasis**

Xueying Fan^1,2^, Baoting Li^1^, Fan Zhang^1^, Meng Liu^1^, Hiu-Yee Kwan^3,4,5*^, Zhongqiu Liu^1,2*^, Tao Su^1,2*^

**This file includes:**

Figure S1 to S5

Table S1 to S2

**
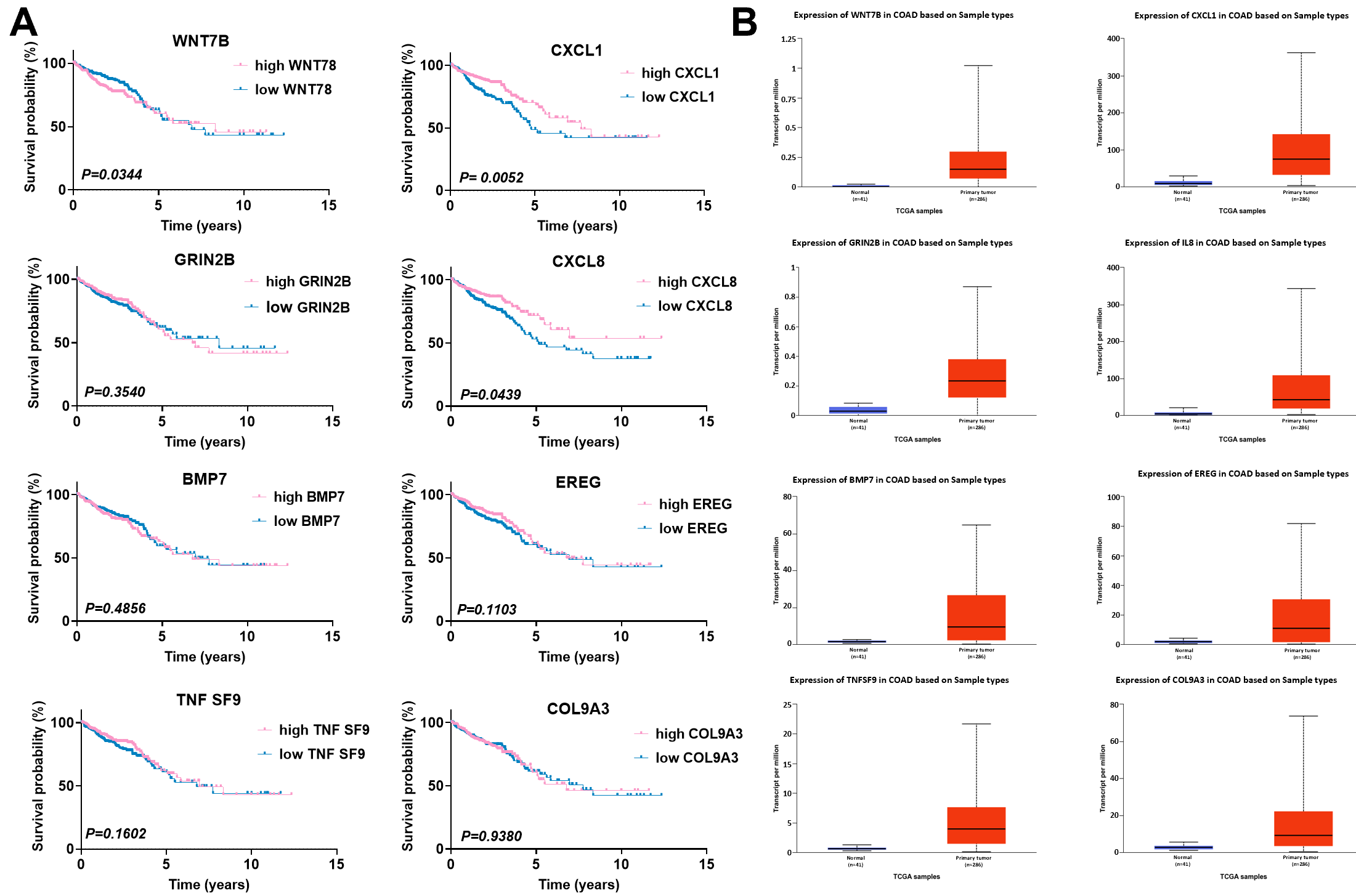
**

**Supplementary Figure S1. Relationship between the differentially expressed genes (DEGs) and CRC patient survival.**

**A)** Correlation analysis between DEGs and CRC patient survival. **B)** Expression levels of the DEGs from COAD patients in the TCGA database.

**
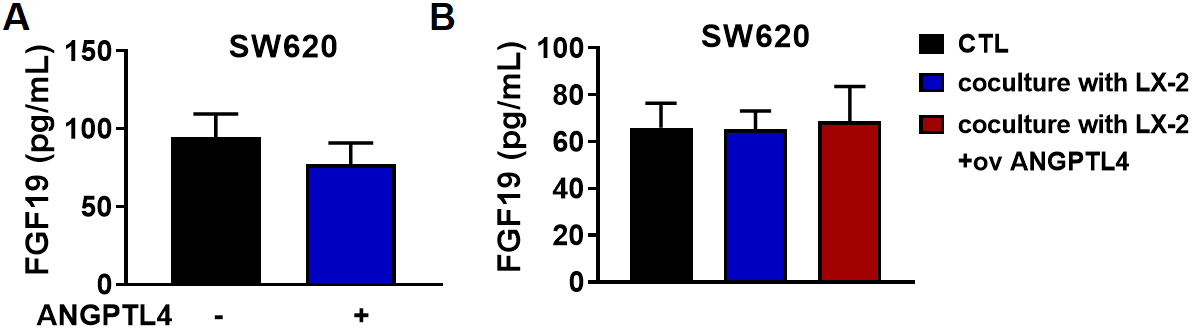
Supplementary Figure S2. Overexpression of ANGPTL4 does not affect the FGF19 release.**

1. The content of FGF19 in the culture medium of recombinant ANGPTL4 protein-treated SW620 was detected using the ELISA assay. **B) S**W620 coculture with LX-2 cells overexpressing or not overexpressing of ANGPTL4, and then the FGF19 content in the coculture medium was detected using the ELISA assay. Data are shown as Mean ± SD, n=3.

**
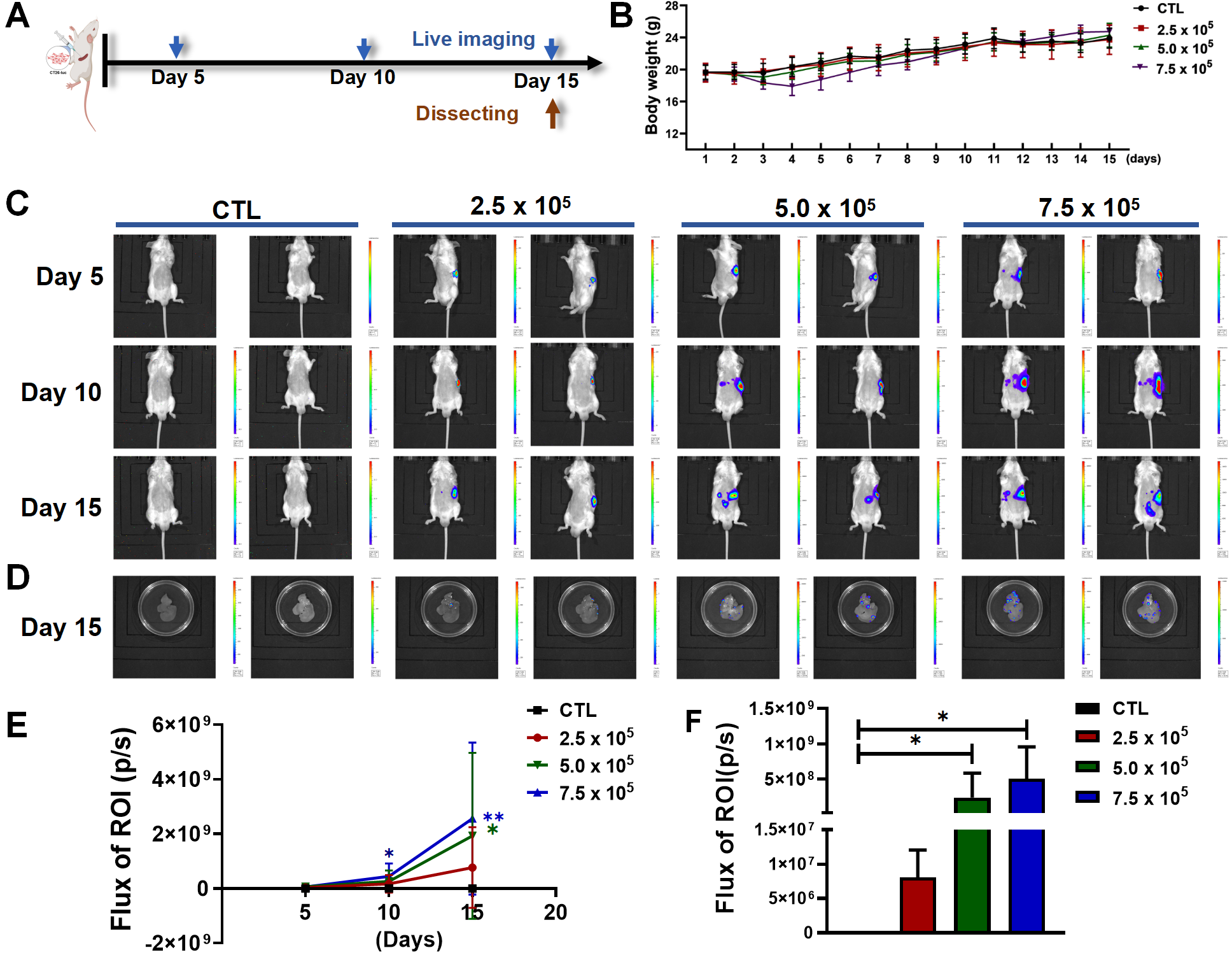
**

**Supplementary Figure S3. The established mouse models with different degrees of CRCLM.**

**A)** Timeline for establishing a mouse CRCLM model. Varying cell numbers of CT26-luc cells were inoculated into the spleen of BALB/c mice. Mice were randomly divided into 4 groups, including Control (CTL), 2.5×10^5^ cells/mice, 5.0×10^5^ cells/mice, 7.5×10^5^ cells/mice. **B)** The body weight of the mice in each group. **C)** Representative live-animal imaging images of mice with tumors. **D)** Representative images of the livers. **E-F)** The quantitative results of the mice fluorescence intensity **(E)** and the livers fluorescence intensity **(F)** were analyzed using the Living Image software 4.4. Data are shown as Mean ±SD, n=6.


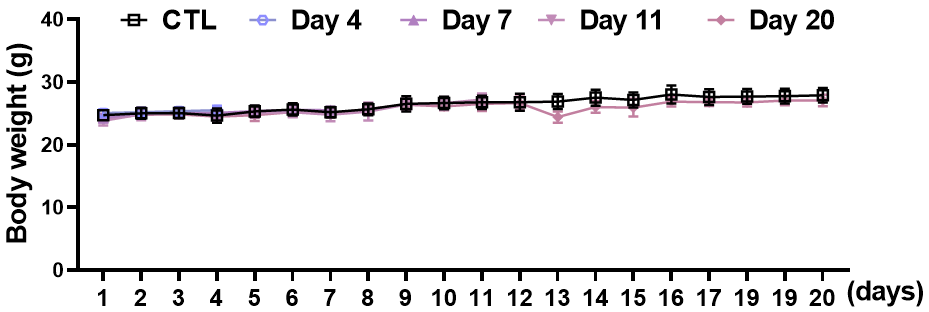


**Supplementary Figure S4.** The body weight of the mice in different stages of CRCLM in each group (n=6).


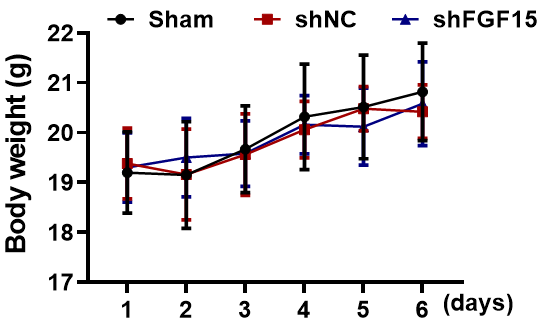


**Supplementary Figure S5.** The body weight of the mice injected with CT26-luc-shNC cells or CT26-luc-shFGF15 cells in each group (n=6).

**Supplementary Table S1. Clinical sample information of tissue microarray.**

| **Characteristic** | **CRC,**  **n = 38** | **Normal, n = 4** |
| --- | --- | --- |
| Age | (36, 82) | NA |
| 36-45 | 5(13.2%) | 0 |
| 46-82 | 28(73.7%) | 0 |
| Unknown | 5(13.1%) | 4(100%) |
| Gender |  |  |
| Female | 18 (47.4%) | 0 |
| Male | 20 (52.6%) | 4 (100%) |
| Tissue Source |  |  |
| healthy population | 0 | 4 (100%) |
| colon adenoma | 6 (15.8%) | 0 |
| colorectal cancer with no metastasis | 21 (55.3%) | 0 |
| colorectal cancer liver metastasis | 8 (21.1%) | 0 |
| colorectal cancer pulmonary metastasis | 1 (2.6%) | 0 |
| colorectal cancer ovarian metastasis | 2 (5.2%) | 0 |
| Tissue Type |  |  |
| normal colon mucosa | 0 | 4 (100%) |
| colon adenoma | 6 (15.8%) | 0 |
| primary tumor | 13 (34.2%) | 0 |
| adjacent tissues | 13 (34.2%) | 0 |
| distant tissues | 5 (13.2%) | 0 |
| liver metastases | 3 (7.9%) | 0 |
| pulmonary metastases | 1 (2.6%) | 0 |
| ovarian metastases | 2 (5.3%) | 0 |
| negative lymph nodes | 5 (13.2%) | 0 |
| positive lymph nodes | 8 (21.1%) | 0 |
| AJCC Cancer Staging Manual, 7th Edition |  |  |
| — | 6 (15.8%) | 4 (100%) |
| 1 | 2 (5.3%) | 0 |
| 2 | 7 (18.4%) | 0 |
| 3 | 12 (31.6%) | 0 |
| 4 | 11 (28.9%) | 0 |

**Supplementary Table S2.** **Primers for quantitative real-time PCR.**

| **Species** | **Gene name** |  | **Primers** |
| --- | --- | --- | --- |
| Mouse | FAP | forward | 5’- GTCACCTGATCGGCAATTTGT - 3’ |
|  |  | reverse | 5’- CCCCATTCTGAAGGTCGTAGAT - 3’ |
| Mouse | GAPDH | forward | 5’- AGGTCGGTGTGAACGGATTTG - 3’ |
|  |  | reverse | 5’- GGGGTCGTTGATGGCAACA - 3’ |
